# Supplementary material for: Antibacterial and Antiallergic Effects of Three Tea Extracts on Histamine-Induced Dermatitis
Source: Pharmaceuticals (Basel). 2024 Sep 7;17(9):1181. doi: 10.3390/ph17091181 (PMC11435320; doi:10.3390/ph17091181)
Supplement: Supplementary file 1 [file pharmaceuticals-17-01181-s001.zip › pharmaceuticals-3143822-supplementary.pdf]

Table S1. Chemical components of Keemun black tea, Hangzhou green tea, and Fujian white tea water extract by UPLC-Q-TOF.

NO	Identity	RT [min]	Measured mass(m/z)	Formula	Relative content%		
					LC	HC	BC
1	L-Glutamic acid	0.922	147.05287	$C_5H_9NO_4$	1.005	0.363	0.473
2	Choline	0.933	103.09989	$C_5H_{13}NO$	1.101	1.363	0.726
3	α,α-Trehalose	0.942	342.11622	$C_{12}H_{22}O_{11}$	2.338	0.438	0.486
4	L-Valine	0.971	117.07899	$C_5H_{11}NO_2$	0.382	0.266	0.265
5	Gluconic acid	0.972	196.05806	$C_6H_{12}O_7$	0.113	0.794	0.209
6	Methanesulfonic acid	0.978	95.98792	CH_4O_3S	0.011	0.007	0.011
7	5-Hydroxymethyl-2-furaldehyde	0.981	126.03163	$C_6H_6O_3$	0.144	0.068	0.050
8	D-(-)-Quinic acid	0.982	192.06323	$C_7H_{12}O_6$	0.687	3.421	3.274
9	Pipecolic acid	0.983	129.07886	$C_6H_{11}NO_2$	0.314	0.092	0.158
10	D-(+)-Pyroglutamic Acid	1.052	129.04247	$C_5H_7NO_3$	0.761	0.449	0.775
11	Citric acid	1.078	192.02703	$C_6H_8O_7$	0.784	0.792	2.308
12	Nicotinamide	1.08	122.04798	$C_6H_6N_2O$	0.141	0.127	0.087
13	Succinic acid	1.242	118.02647	$C_4H_6O_4$	0.033	0.024	0.035
14	Adenosine	1.259	267.09582	$C_{10}H_{13}N_5O_4$	0.064	0.064	0.054
15	L-Norleucine	1.394	131.09454	$C_6H_{13}NO_2$	0.587	0.303	0.518
16	L-Phenylalanine	2.244	165.07867	$C_9H_{11}NO_2$	0.430	0.149	0.544
17	Gentisoyl glucoside	2.433	316.07956	$C_{13}H_{16}O_9$	0.026	0.071	0.379
18	2,3-Dihydroxybenzoic acid	2.866	154.02644	$C_7H_6O_4$	0.009	0.035	0.015
19	Theobromine	3.403	180.06439	$C_7H_8N_4O_2$	0.268	0.686	1.249
20	4-methyl-7-[(2R,3S,4S,5S,6R)-3,4,5-trihydroxy-6-(hydroxymethyl)oxan-2-yl]oxychromen-2-one	6.934	338.1	$C_{16}H_{18}O_8$	0.026	0.071	0.379
21	(-)-Gallocatechin	7.157	306.07345	$C_{15}H_{14}O_7$	8.040	0.008	4.968
22	Caffeine	9.965	194.07998	$C_8H_{10}N_4O_2$	54.654	60.633	56.720
23	Esculetin	11.179	178.02626	$C_9H_6O_4$	0.043	0.295	0.032
24	(4S)-4-hydroxy-3,5,5-trimethyl-4-[(1E)-3-{[(2R,3R,4S,5S,6R)-3,4,5-trihydroxy-6-(hydroxymethyl)oxan-2-yl]oxy}but-1-en-1-yl]cyclohex-2-en-1-one	12.175	386.19309	$C_{19}H_{30}O_8$	0.101	-	0.010
25	Phenylethyl 2-Glucoside	12.702	284.12516	$C_{14}H_{20}O_6$	0.031	0.008	0.031
26	Epigallocatechin gallate	12.712	458.08481	$C_{22}H_{18}O_{11}$	5.560	0.253	10.674
27	7-hydroxy-6-methoxy-2H-chromen-2-one	12.787	192.04196	$C_{10}H_8O_4$	0.003	0.021	-

28	PEG n8	12.834	370.21938	$C_{16}H_{34}O_9$	0.066	0.066	0.035
29	Vicenin II	12.962	594.15772	$C_{27}H_{30}O_{15}$	0.057	0.066	0.041
30	Salicylic acid	13.534	138.03149	$C_7H_6O_3$	0.094	0.044	0.100
31	(2R,3R,4S,5S,6R)-2-[(3Z)-hex-3-en-1-yloxy]-6-(hydroxymethyl)oxane-3,4,5-triol	13.612	262.14092	$C_{12}H_{22}O_6$	0.028	0.013	0.016
32	Vicenin III	13.711	564.14715	$C_{26}H_{28}O_{14}$	0.329	0.563	0.257
33	4-(4-hydroxy-2,6,6-trimethyl-3-{[(2R,3R,4S,5S,6R)-3,4,5-trihydroxy-6-(hydroxymethyl)oxan-2-yl]oxy}cyclohex-1-en-1-yl)butan-2-one	13.853	388.20876	$C_{19}H_{32}O_8$	0.121	0.001	0.009
34	myricetin 3-O-beta-D-galactopyranoside	13.902	480.08991	$C_{21}H_{20}O_{13}$	0.427	0.065	0.271
35	Apigenin-4 '- glucoside	14.221	432.10503	$C_{21}H_{20}O_{10}$	0.046	0.085	0.054
36	Vitexin rhamnoside	14.36	578.16279	$C_{27}H_{30}O_{14}$	0.148	0.100	0.060
37	PEG n11	14.425	502.29807	$C_{22}H_{46}O_{12}$	0.016	0.034	0.010
38	Morin	14.636	302.04181	$C_{15}H_{10}O_7$	0.201	0.158	0.113
39	Isovitexin	14.683	432.10491	$C_{21}H_{20}O_{10}$	0.043	0.087	0.076
40	4-Hydroxybenzaldehyde	14.74	122.0368	$C_7H_6O_2$	0.049	0.005	0.093
41	Gallic acid	14.771	170.02037	$C_7H_6O_5$	0.007	-	0.011
42	Isoquercitrin	14.884	464.09494	$C_{21}H_{20}O_{12}$	0.585	0.380	0.295
43	Rutin	14.888	610.15276	$C_{27}H_{30}O_{16}$	1.250	0.247	0.221
44	Quercetin	14.889	302.04185	$C_{15}H_{10}O_7$	0.049	0.016	0.012
45	Grosvenorine	15.585	740.21585	$C_{33}H_{40}O_{19}$	0.006	0.026	0.063
46	Astragalin	15.648	448.1001	$C_{21}H_{20}O_{11}$	0.187	0.096	0.313
47	Kaempferol	15.651	286.04693	$C_{15}H_{10}O_6$	0.112	0.059	0.175
48	Kaempferol-3-O-rutinoside	15.657	594.15805	$C_{27}H_{30}O_{15}$	0.249	0.143	0.243
49	(2R,3R,4S,5S,6R)-2-{[(2E,6R)-6-hydroxy-2,6-dimethylocta-2,7-dien-1-yl]oxy}-6-(hydroxymethyl)oxane-3,4,5-triol	15.749	332.18263	$C_{16}H_{28}O_7$	0.246	0.004	0.028
50	4,8-dihydroxy-6,6,8-trimethyl-1H,3H,4H,4aH,5H,6H,7H,7aH,8H,9H-azuleno[5,6-c]furan-1-one	15.833	266.14873	$C_{15}H_{22}O_4$	0.074	0.008	0.018
51	(2R,3S,4S,5R,6R)-2-(hydroxymethyl)-6-[4-(4-hydroxy-2,6,6-trimethylcyclohexen-1-yl)butan-2-yloxy]oxane-3,4,5-triol	15.922	374.22959	$C_{19}H_{34}O_7$	0.246	0.004	0.028
52	Isophorone	16.232	138.10425	$C_9H_{14}O$	0.023	0.021	0.017

53	2-Hydroxy-3-(5-hydroxy-7,8-dimethoxy-4-oxo-4H-chromen-2-yl)phenyl β-D-glucopyranoside	16.391	492.12593	$C_{23}H_{24}O_{12}$	0.025	0.007	0.004
54	Afzelin	16.45	432.1051	$C_{21}H_{20}O_{10}$	0.005	0.017	0.010
55	Fisetin	16.457	286.04688	$C_{15}H_{10}O_{6}$	0.003	0.007	0.004
56	Tiliroside	17.166	594.13684	$C_{30}H_{26}O_{13}$	0.002	0.010	0.033
57	Celerioside E	17.579	418.25592	$C_{21}H_{38}O_{8}$	0.081	0.025	0.062
58	Luteolin	17.781	286.04742	$C_{15}H_{10}O_{6}$	0.009	0.040	0.009
59	6,8-dihydroxy-3-(10-hydroxyundecyl)-3,4-dihydro-1H-2-benzopyran-1-one	18.825	350.20604	$C_{20}H_{30}O_{5}$	0.016	0.028	0.010
60	Corchorifatty acid F	18.828	328.22506	$C_{18}H_{32}O_{5}$	0.014	0.048	0.016
61	6-hydroxy-3,5a,9-trimethyl-2H,3H,3aH,4H,5H,5aH,6H,7H,9aH,9bH-naphtho[1,2-b]furan-2-one	19.232	250.15386	$C_{15}H_{22}O_{3}$	0.042	0.013	0.010
62	Methoxyacetyl fentanyl	19.407	352.22175	$C_{22}H_{28}N_{2}O_{2}$	0.014	0.017	0.012
63	Bis(4-ethylbenzylidene)sorbitol	20.102	414.20329	$C_{24}H_{30}O_{6}$	0.850	1.378	0.775
64	Glabridin	20.506	324.1356	$C_{20}H_{20}O_{4}$	0.016	-	-
65	Carnosic Acid	20.754	332.1956	$C_{20}H_{28}O_{4}$	0.009	0.014	0.005
66	Dibutyl Sebacate	20.804	314.24587	$C_{18}H_{34}O_{4}$	0.119	0.134	0.088
67	Bicyclo Prostaglandin E2	20.872	334.21122	$C_{20}H_{30}O_{4}$	0.046	0.044	0.022
68	(±)9-HpODE	21.124	312.23027	$C_{18}H_{32}O_{4}$	0.002	0.002	0.002
69	Bempedoic Acid	21.264	344.25547	$C_{19}H_{36}O_{5}$	0.011	0.016	0.008
70	9-Oxo-10(E),12(E)-octadecadienoic acid	22.043	294.21877	$C_{18}H_{30}O_{3}$	0.060	0.103	0.039
71	Monolaurin	22.113	274.21374	$C_{15}H_{30}O_{4}$	0.017	0.024	0.014
72	5-OxoETE	22.342	318.21647	$C_{20}H_{30}O_{3}$	0.005	0.064	0.033
73	(3beta,9xi)-3-(beta-D-Glucopyranosyloxy)-14-hydroxycard-20(22)-enolide	22.72	536.2954	$C_{29}H_{44}O_{9}$	-	0.008	0.011
74	2,3-dihydroxypropyl 12-methyltridecanoate	23.053	302.24482	$C_{17}H_{34}O_{4}$	0.537	0.813	0.342
75	TOFA	23.056	324.22701	$C_{19}H_{32}O_{4}$	0.467	0.706	0.285
76	Linoleoyl Ethanolamide	23.244	323.28161	$C_{20}H_{37}NO_{2}$	0.018	0.024	0.014
77	2,2'-Methylenebis(4-methyl-6-tert-butylphenol)	23.422	340.24028	$C_{23}H_{32}O_{2}$	3.154	3.223	1.711
78	4-tert-Amylphenol	23.423	164.11852	$C_{11}H_{16}O$	0.023	0.026	0.012
79	Oleanolic acid	23.634	456.35933	$C_{30}H_{48}O_{3}$	0.022	-	-
80	Linoleic acid	23.916	280.24019	$C_{18}H_{32}O_{2}$	0.004	0.006	0.003
81	Monoolein	24.001	356.29175	$C_{21}H_{40}O_{4}$	0.203	0.350	0.152

82	Stearamide	24.171	283.28694	$C_{18}H_{37}NO$	1.892	2.633	0.812
83	1,2-Dipalmitoylphosphatidylglycerol	24.197	722.51659	$C_{38}H_{75}O_{10}P$	0.126	0.145	0.075
84	Oleoyl ethylamide	24.385	309.30232	$C_{20}H_{39}NO$	0.412	0.392	0.186
85	Elaidic acid	24.399	282.25582	$C_{18}H_{34}O_2$	0.036	0.053	0.026
86	1-Stearoylglycerol	24.426	358.30741	$C_{21}H_{42}O_4$	9.069	14.402	7.212
87	Palmitic acid	24.426	256.24022	$C_{16}H_{32}O_2$	0.003	0.004	0.002
88	Tridemorph	24.453	297.3024	$C_{19}H_{39}NO$	0.118	0.114	0.058
89	Stearic acid	24.86	284.27155	$C_{18}H_{36}O_2$	0.003	0.002	0.002
90	Erucamide	25.13	337.33354	$C_{22}H_{43}NO$	1.339	1.453	-
91	Docosanamide	25.679	339.34927	$C_{22}H_{45}NO$	0.128	0.072	0.050
Amino acid compound/%					3.276	2.324	2.784
Phenols compound/%					3.423	3.452	1.938
Flavonoids compound/%					17.405	2.450	17.954
Alkaloid compounds/%					54.921	61.319	57.969
Polysaccharides and glycosides/%					3.245	1.895	1.671
Organic acid compounds/%					1.844	4.385	5.802
Fatty compound/%					13.471	19.838	8.635
Total identified/%					97.585	95.663	99.753
